# Analysis of the surveillance results of avian influenza in the external environment of huzhou city from 2017 to 2023

**DOI:** 10.1371/journal.pone.0326382

**Published:** 2025-07-03

**Authors:** Yuda Wang, Jianyong Shen, Guangtao Liu, Ziqi Jiang, Hang Liu, Tao Yu, Zhuoxin Ying, Junfen Lin, Yan Liu

**Affiliations:** 1 Huzhou Center for Disease Control and Prevention, Huzhou, Zhejiang, China; 2 Zhejiang Province Infectious Disease Surveillance and Early Warning Professional Backbone Training Project, Hangzhou, Zhejiang, China,; 3 Deqing county Center for Disease Control and Prevention, Huzhou, Zhejiang, China; 4 Anji county Center for Disease Control and Prevention, Huzhou, Zhejiang, China; 5 Linping districy Center for Disease Control and Prevention, Hangzhou, Zhejiang, China; 6 Zhejiang Provincial Center for Disease Control and Prevention, Zhejiang, China; 7 Luqiao districy Center for Disease Control and Prevention, Taizhou, Zhejiang, China; The University of Hong Kong, CHINA

## Abstract

**Background:**

Avian influenza is an infectious disease of birds caused by the influenza A virus, which can infect a variety of domestic,wild birds and even cross the species barrier and infect humans.To understand the contamination of avian influenza virus in the external environment of poultry in Huzhou City from 2017 to 2023 and to assess the risk of human infection with avian influenza.

**Methods:**

A total of 3,400 environmental specimens from five types of venues in Huzhou City were collected and tested for influenza A virus nucleic acid using fluorescent reverse transcription polymerase chain reaction (RT-PCR).

**Results:**

From 2017 to 2023, with 15.44% overall positive rate of influenza A virus. The predominant subtype of avian influenza virus was H9 (accounting for 54.67%). The peak of positive influenza virus detection rates occurred in winter and spring seasons every year. The venue with highest positive rate was poultry slaughtering and processing plants (41.83%), followed by urban and rural live poultry markets (35.48%); among all types of specimens, the highest positive rate was detected in swab specimens from the surfaces of poultry slaughtering or display tables (47.37%), followed by wastewater from poultry washing (45.83%), and surfaces of cages (27.65%).

**Conclusion:**

The contamination of avian influenza virus in the poultry environment in Huzhou City is relatively severe, with diverse subtypes. There is a potential risk of human infection with avian influenza virus, and real-time monitoring of avian influenza virus in the poultry environment needs to be strengthened.

## Introduction

Avian influenza is an infectious disease of birds caused by the influenza A virus, which can infect a variety of domestic and wild birds [1]. According to the severity of pathogenicity, avian influenza viruses can be divided into highly pathogenic, low pathogenic, and non-pathogenic types [2,3]. The highly pathogenic avian influenza viruses that currently cause human infection are mainly the H5 and H7 subtypes. Infections with highly pathogenic avian influenza (H5N1, H5N6) and H7N9 avian influenza in humans can easily lead to symptoms such as shortness of breath, severe pneumonia, and multiple organ dysfunction, with a high mortality rate. They are classified as Class B infectious diseases in China [4–6]. In recent years, outbreaks of avian influenza of different subtypes have occurred in multiple countries and regions, killing hundreds of millions of domestic and wild birds. At the same time, avian influenza viruses continue to undergo genetic recombination within the host to produce new subtypes, some of which can cross the species barrier and infect humans, posing a serious threat to human health and public health [7,8]. Previous study showed that contact with live poultry or exposure to live poultry markets is the most important risk factor for human infection with avian influenza virus [9]. High-frequency contact between humans and poultry may increase the risk of recombination and cross-species transmission of avian influenza virus. As an important region in the eastern part of China, Huzhou City not only has abundant water resources and ecological diversity, but is also an central area for poultry breeding and live poultry trading. Therefore, strengthening external environment monitoring of avian influenza is crucial. This study conducted the external environmental monitoring of avian influenza in Huzhou during 2017–2023,to further understand the distribution of avian influenza viruses in the poultry environment, and to provide a basis for the prevention and control measures of avian influenza and human infection with avian influenza.

## Materials and methods

### Specimen collection

In accordance with the requirements of the “Zhejiang Province Occupational Exposure Population Serology and Environmental Highly Pathogenic Avian Influenza Monitoring Program,” from 2017 to 2023, the Disease Prevention and Control Centers of the three counties (surrounding urban areas) and two districts (central urban areas) in the city carried out environmental monitoring of poultry in urban and rural areas. Each monitoring site included 3–4 sampling points, and 1–2 environmental specimens of poultry were collected from each monitoring point every month. When collecting environmental specimens, a disposable sampling swab was used to pick up a certain amount of fresh poultry feces, or to smear the surface of poultry cages, cutting boards, etc., and then the swab was placed into a 15 ml screw-cap centrifuge tube containing sampling fluid. This study does not involve any animal experiments or clinical trials, hence there are no ethical disputes related to it. Raw data are shown in S1 Table.

### Laboratory test

Reverse transcription polymerase chain reaction (RT-PCR) was used to detect avian influenza virus nucleic acid in the collected environmental specimens of poultry. Primers and probes for H5, H7, and H9 avian influenza viruses were provided by the Zhejiang Provincial Center for Disease Control and Prevention. A 200 μl sample liquid was taken, and nucleic acid was extracted using the NP968 automatic nucleic acid extractor (Tianlong Technology Co., Ltd., Xi’an, China). The TaKaRa One Step RNA PCR Kit (batch number: AK6001) was used for one-step real-time reverse transcription PCR (real-time RT-PCR) amplification, and amplification was performed using the 7500 fluorescent quantitative PCR instrument (ABI Company, USA). The reaction system was 25 μl, and the system configuration was carried out according to the instructions of the kit.

### Statistical analysis

Statistical analysis was performed using IBM SPSS 25.0 and Excel software. The comparison of rates was performed using the chi-square test, and differences were considered statistically significant when *p* < 0.05.

## Results

### Detection of influenza virus subtype nucleic acid in poultry external environmental specimens

Out of 3,400 specimens, 525 were positive for influenza A virus nucleic acid, with a positive rate of 15.44%. The positive rate for avian influenza virus from 2017 to 2023 ranged from 9.91% to 20.20%, with the highest positive rate in 2019 at 20.20% and the lowest in 2022 at 9.91%. The difference in positive rates across different years was statistically significant (*χ*^*2*^ = 22.213, *p *< 0.001). Among the 525 positive specimens, 287 were positive for H9 (accounting for 54.67%), 34 for H7 (6.48%), 24 for H5 (4.57%), 31 were mixed positives, and 212 were positives for other untyped subtypes (Table 1).

**Table 1:** Detection of influenza virus subtypes nucleic acid from 2017 to 2023.

Years	Specimen Count	H5		H7		H9		Influenza A Virus without Subtype Specification		The number of positive cases for two or more types		Influenza A Viruses Total	
		Positive Count	Positive Rate(%)	Positive Count	Positive Rate(%)	Positive Count	Positive Rate(%)	Positive Count	Positive Rate(%)	Positive Count	Positive Rate(%)	Positive Count	Positive Rate(%)
**2017**	825	5	0.61	23	2.79	57	6.91	62	7.52	14	1.70	132	16.00
**2018**	817	4	0.49	5	0.61	44	5.39	58	7.10	3	0.37	108	13.22
**2019**	510	7	1.37	5	0.98	70	13.73	30	5.88	9	1.76	103	20.20
**2020**	293	0	0.00	0	0.00	30	10.24	21	7.17	0	0.00	51	17.41
**2021**	299	4	1.34	1	0.33	28	9.36	14	4.68	1	0.33	46	15.38
**2022**	343	3	0.88	0	0.00	18	5.26	15	4.39	3	0.88	33	9.91
**2023**	313	1	0.32	0	0.00	40	12.78	12	3.83	1	0.32	52	16.61
**Total**	3400	24	0.71	34	1.00	287	8.44	212	6.24	31	0.91	525	15.44

### Temporal distribution of influenza a virus positivity in poultry external environmental specimens

**Fig 1:**
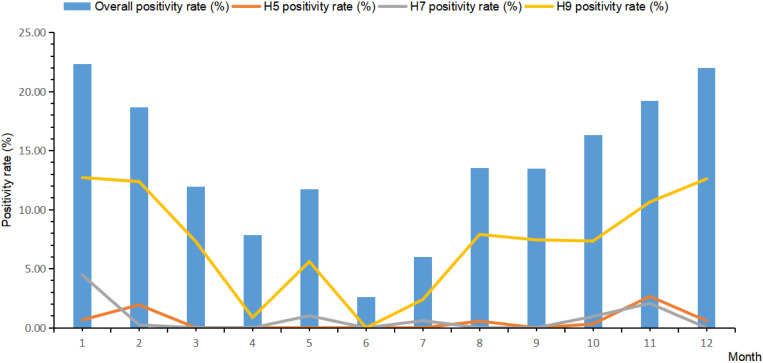
The monthly average positive rate and trend of avian influenza virus in Huzhou city from 2017 to 2023.

The positivity rate in poultry external environmental specimens varies over time, showing a distinct seasonal pattern. The average monthly positivity rate throughout the year generally exhibits a “U” shaped distribution. The peak periods for the detection of influenza A virus nucleic acid are during the winter and spring seasons each year, specifically forming a peak from October to February of the following year, while the positivity rate is relatively lower in the summer season. The seasonal positive rates of different subtypes are the same, especially for the H9 subtype, where the seasonality is most pronounced (Fig 1).

### Regional distribution of positive specimens in the external environment of poultry

From 2017 to 2023, the positivity rate of influenza A virus nucleic acid is 7.02% (86/1,225) and 19.91% (439/2,175) in the central urban area and surrounding urban area of Huzhou City, respectively. The H5, H7 and H9 positivity rates were 0%, 0.90%, and 3.51% in the central urban area, while in surrounding urban area, they are 1.10%, 1.06%, and 10.27%, respectively. The positivity rates for Influenza A, H5 and H9 subtypes in the external environment of poultry in the surrounding urban areas were significantly higher than those in the central urban area (*χ*^*2*^ = 103.997, 13.613, 60.245, *P* < 0.001) (Table 2).

**Table 2:** Regional Distribution of Positive Specimens in the External Environment of Poultry in Huzhou City from 2017 to 2023.

Avian Influenza Virus Subtypes	Central City District		Surrounding Urban District		*χ* ^2^	*P*
	Positive Count	Positive Rate (%)	Positive Count	Positive Rate (%)		
Influenza A Viruses	86	7.02	439	19.91	103.997	0.000
H5	0	0	24	1.10	13.613	0.000
H7	11	0.90	23	1.06	0.201	0.772
H9	43	3.51	244	10.27	60.245	0.000

### Distribution of positive specimens in the external environment of poultry by monitoring sites

The positive rates for Influenza A virus nucleic acid detected in environmental specimens from different monitoring sites were as follows: the highest positive rate was found in poultry slaughtering and processing plants, reaching 41.83% (325/777), followed by urban and rural live poultry markets at 35.48% (165/465), large-scale poultry farms (households) at 2.64% (25/946), areas with backyard poultry farmers at 1.12% (10/895), and no positives were detected in the wild migratory bird habitats. The overall difference in the positive rates of Influenza A virus across different monitoring sites was statistically significant (*χ*^*2*^ = 874.59, *P* < 0.001) (Table 3).

**Table 3:** The distribution of positive specimen sites detected in the external environment of Huzhou City between 2017 and 2023.

Location or Site	Specimen count	positive count	positive rate (%)
Urban and Rural Live Poultry Markets	465	165	35.48
Scaled Poultry Farms (Households)	946	25	2.64
Areas Concentrated with Free-range Poultry Households	895	10	1.12
Poultry Slaughtering and Processing Plants	777	325	41.83
Habitats of Migratory Wild Birds	317	0	0.00
***χ*** ^2^	874.59		
*P*	<0.01		

### Detection of influenza a virus nucleic acid positivity in different types of environmental specimens

From 2017 to 2023, a total of 2,583 fecal specimens, 217 cage surface swab specimens, 231 poultry drinking water samples, 114 cutting or display board surface swab specimens of slaughtered or displayed poultry, 48 poultry washing wastewater samples, and 207 other types of specimens were collected. The positive rates for different types of specimens were as follows: swab specimens from the surfaces of cutting or display boards for poultry meat (47.37%), poultry washing wastewater (45.83%), cage surface swab specimens (27.65%), poultry drinking water (12.99%), fecal specimens (11.54%), and other types of specimens had a positivity rate of 29.47%. The overall difference in the positive rates of Influenza A virus across different specimen types was statistically significant (*χ*^*2*^ = 210.14, *P* < 0.001) (Table 4).

**Table 4:** Distribution of positive specimen types detected in the external environment of Huzhou city from 2017 to 2023 (%).

Specimen Type	2017	2018	2019	2020	2021	2022	2023	Total
**Cages Surface Swab Specimen**	31.9(22/69)	37.5(12/32)	41.2(7/17)	23.1(3/13)	25.0(5/20)	14.3(5/35)	19.4(6/31)	27.65 (60/217)
**Slaughter or Poultry Meat Cutting Board Surface Swab Specimen**	45.5(5/11)	100(2/2)	63.6(7/11)	69.6(16/23)	19.2(5/26)	55.6(10/18)	39.1(9/23)	47.37 (54/114)
**Fecal Specimen**	15.1(98/651)	10.7(73/682)	14.6(60/411)	12.1(27/224)	9.9(21/212)	2.5(5/205)	7.1(14/198)	11.54 (298/2583)
**Sewage from Poultry Washing**	20.0(1/5)	53.8(7/13)	87.5(7/8)	20.0(1/5)	50.0(2/4)	27.3(3/11)	50.0(1/2)	45.83 (22/48)
**Poultry Drinking Water**	1.9(1/54)	2.6(1/38)	0(0/18)	26.7(4/15)	20.8(5/24)	7.0(3/43)	38.5(15/39)	12.99 (30/231)
**Others**	14.3(5/35)	2.6(13/50)	48.9(22/45)	0(0/13)	61.5(8/13)	22.6(7/31)	30.0(6/20)	29.47 (61/207)
**Total**	16.0(132/825)	13.2(108/817)	20.2(103/510)	17.4(51/293)	15.4(46/299)	9.6(33/343)	16.6(52/313)	15.44 (525/3400)
***χ*** ^2^	210.14							
*P*	<0.01							

## Discussion

This study indicated that avian influenza viruses in Huzhou City can be detected throughout the year, showing an obvious seasonal characteristic of being higher in winter and spring than in summer and autumn. Additionally, According to the monitoring data over the years, the 27 H7N9 cases reported in Huzhou City since 2013 were also concentrated in the winter and spring. Some studies have found that October to March each year is the optimal survival season for avian influenza viruses [10], and low-temperature conditions are more conducive to proliferation and spread of avian influenza viruses [11]. This suggests that relevant departments should take preventive and control measures in advance before the arrival of the epidemic season. From 2017 to 2023, each subtype of avian influenza virus was detected in the external environmental specimens of Huzhou City, with the H9 subtype being predominant, followed by untyped influenza A, and the H5 and H7 subtypes and mixed types accounting for a lower proportion, which is consistent with the monitoring situations in Changzhou, Quzhou, Ma’anshan, and other regions [12–16]. From 2017 to 2023, Huzhou City only reported one case of human infection with H7N9 avian influenza in January and March 2017, and no cases of human infection with avian influenza have occurred since then. However, unclassified influenza A subtypes still need to be vigilant, which also pose a risk of infecting humans. It is suggested that the next step should be to strengthen further typing and detection of untyped viruses.

Live poultry markets were shown to be one of the independent risk factors for human infection with highly pathogenic avian influenza viruses [9]. An epidemiological study of human H7N9 avian influenza cases in Huzhou in 2013 showed that among all H7N9 cases, patients with a history of exposure to live poultry markets accounted for as high as 75.00% [17]. This indicates that live poultry markets are important environmental risk factors for human infection with H7N9 avian influenza, which is consistent with the survey results in neighboring cities. In this study, the positive rate of influenza A virus in the environment of urban and rural live poultry markets and poultry slaughtering and processing plants was much higher than that of local backyard poultry farmers and large-scale poultry farms, which may be related to the diversity of sources of live poultry markets and slaughtering and processing plants. The extensive sources also provide more possibilities for the cross-city and cross-province spread of the virus. Since July 2014, the central urban area of Huzhou City has banned live poultry transactions and closed all live poultry markets. Therefore, in this study, the positive rate in the central urban area was significantly lower than that in the surrounding urban areas, indirectly proving that restricting live poultry transactions can effectively reduce the spread of avian influenza viruses, thus also lowering the risk of humans being infected with avian influenza.

Existing data show that there is an accumulation effect of avian influenza virus pollution in the wastewater of live poultry markets and the surfaces of poultry meat boards [18], indicating that under the existing slaughtering methods of poultry slaughtering and processing plants, the opportunity for the spread of avian influenza viruses has greatly increased, which also increases the risk of human infection with avian influenza viruses. Although in recent years, relevant documents have been issued to regulate the poultry trading process, including policies such as “cleaning once a day, disinfection once a week, and a market rest day once a month,” some poultry trading stalls still cannot meet the cleaning and disinfection measures as required.

In summary, the agricultural departments, health departments, and market supervision departments of Huzhou City should strengthen cooperation to further prevent and control avian influenza viruses and human infections with avian influenza. For live poultry markets and processing plants with high positive rates of avian influenza viruses, unified disinfection should be carried out, and partial closed management should be carried out when necessary; strengthen the supervision of the source of poultry in live poultry markets and poultry slaughtering and processing plants to prevent the inflow of sick poultry; strengthen the supervision and training of personnel engaged in poultry-related work, and strengthen personal protection, standardize the killing and cleaning of poultry and other related operations; further strengthen the monitoring of different subtypes of avian influenza, at the same time, conduct pathogenicity testing on H7 positive specimens to distinguish whether they are highly pathogenic H7 viruses; regularly collect blood samples from key populations for related antibody testing.

This study also has limitations in cross-species research. In the follow-up, we will collect more samples from different bird species and analyze the epidemiological characteristics of different viral subtypes in these species, such as infection rates and viral shedding patterns. We will also combine existing human cases of avian influenza to deeply explore the risk factors of different viral subtypes spreading through different birds and infecting humans, such as the frequency of contact between birds and humans and adaptive changes of the virus across species.

## Supporting information

S1 Table Avian influenza in the external environment of Huzhou city from 2017 to 2023. (XLSX)

## References

[1] Alexander DJ. A review of avian influenza in different bird species. Vet Microbiol. 2000;74(1–2):3–13. doi: 10.1016/s0378-1135(00)00160-7 10799774

[2] Swayne DE, Suarez DL. Highly pathogenic avian influenza. Rev Sci Tech. 2000;19(2):463–82. doi: 10.20506/rst.19.2.1230 10935274

[3] Umar S, Guerin JL, Ducatez MF. Low Pathogenic Avian Influenza and Coinfecting Pathogens: A Review of Experimental Infections in Avian Models. Avian Dis. 2017;61(1):3–15. doi: 10.1637/11514-101316-Review 28301244

[4] Ke C, Mok CKP, Zhu W, Zhou H, He J, Guan W, et al. Human Infection with Highly Pathogenic Avian Influenza A(H7N9) Virus, China. Emerg Infect Dis. 2017;23(8):1332–40. doi: 10.3201/eid2308.170600 28580899 PMC5547808

[5] Bi Y, Zhang Z, Liu W, Yin Y, Hong J, Li X, et al. Highly Pathogenic Avian Influenza A(H5N1) Virus Struck Migratory Birds in China in 2015. Sci Rep. 2015;5:12986. doi: 10.1038/srep12986 26259704 PMC4531313

[6] Lu S, Zhao Z, Zhang J, Wang W, He X, Yu M, et al. Genetics, pathogenicity and transmissibility of novel reassortant H5N6 highly pathogenic avian influenza viruses first isolated from migratory birds in western China. Emerg Microbes Infect. 2018;7(1):6. doi: 10.1038/s41426-017-0001-1 29362400 PMC5837145

[7] Lee D-H, Criado MF, Swayne DE. Pathobiological Origins and Evolutionary History of Highly Pathogenic Avian Influenza Viruses. Cold Spring Harb Perspect Med. 2021;11(2):a038679. doi: 10.1101/cshperspect.a038679 31964650 PMC7849344

[8] Nuñez IA, Ross TM. A review of H5Nx avian influenza viruses. Ther Adv Vaccines Immunother. 2019;7:2515135518821625. doi: 10.1177/2515135518821625 30834359 PMC6391539

[9] Wang X, Wang Q, Cheng W. Risk factors for avian influenza virus contamination of live poultry markets in Zhejiang, China during the 2015–2016 human influenza season. Scientific Reports. 2017;7(1):42722. 28256584 10.1038/srep42722 PMC5335333

[10] Li J, Rao Y, Sun Q, Wu X, Jin J, Bi Y, et al. Identification of climate factors related to human infection with avian influenza A H7N9 and H5N1 viruses in China. Sci Rep. 2015;5:18094. doi: 10.1038/srep18094 26656876 PMC4676028

[11] Liu Z, Geng X, Cui Z, Li W, Ou X, Liao G. Construction and identification of influenza plasmid pool imparting high yields to candidate vaccine viruses in Vero cell at low temperature. J Cell Mol Med. 2020;24(19):11198–210. doi: 10.1111/jcmm.15672 32902192 PMC7576294

[12] Anzhi Z, Qian Z, a J. Surveillance for avian influenza virus in poultry related environment in Changzhou, Jiangsu, 2016−2022. Disease Surveillance. 2023;39(1):43–7.

[13] Cao G, Zhan B, Chen X. Surveillance of avian influenza virus in population with occupational exposure and in out-environment in Quzhou, Zhejiang. Dis Surveill. 2013;28:884–7.

[14] Huang S, Yang R, Lyu L. Surveillance results of avian influenza virus in external environment in Quzhou, Zhejiang, 2013–2018. Disease Surveillance. 2019;34(9):835–8.

[15] Li L-H, Yu Z, Chen W-S, Liu S-L, Lu Y, Zhang Y-J, et al. Evidence for H5 avian influenza infection in Zhejiang province, China, 2010-2012: a cross-sectional study. J Thorac Dis. 2013;5(6):790–6. doi: 10.3978/j.issn.2072-1439.2013.12.45 24409357 PMC3886696

[16] Li R, Zhang T, Xu J, Chang J, Xu B. Isolation of two novel reassortant H3N6 avian influenza viruses from long-distance migratory birds in Jiangxi Province, China. Microbiologyopen. 2020;9(8):e1060. doi: 10.1002/mbo3.1060 32468676 PMC7424263

[17] Han J, Jin M, Zhang P, Liu J, Wang L, Wen D, et al. Epidemiological link between exposure to poultry and all influenza A(H7N9) confirmed cases in Huzhou city, China, March to May 2013. Euro Surveill. 2013;18(20):20481. 23725866

[18] Islam SS, Akwar H, Hossain MM, Sufian MA, Hasan MZ, Chakma S, et al. Qualitative risk assessment of transmission pathways of highly pathogenic avian influenza (HPAI) virus at live poultry markets in Dhaka city, Bangladesh. Zoonoses Public Health. 2020;67(6):658–72. doi: 10.1111/zph.12746 32558220

